# Adaptation to average duration

**DOI:** 10.3758/s13414-020-02134-8

**Published:** 2020-10-08

**Authors:** Jennifer E. Corbett, Berfin Aydın, Jaap Munneke

**Affiliations:** 1 College of Health, Medical, and Life Sciences, Division of Psychology, Brunel University London, MJ-122, Kingston Lane, London, Uxbridge UB8 3PH UK; 2 Centre for Cognitive Neuroscience, Brunel University London, London, UK; 3 Aysel Sabuncu Brain Research Center and Interdisciplinary Neuroscience Program, Bilkent University, Ankara, Turkey

**Keywords:** Perceptual averaging, Temporal vision, Visual aftereffect

## Abstract

There has been a recent surge of research examining how the visual system compresses information by representing the average properties of sets of similar objects to circumvent strict capacity limitations. Efficient representation by perceptual averaging helps to maintain the balance between the needs to perceive salient events in the surrounding environment and sustain the illusion of stable and complete perception. Whereas there have been many demonstrations that the visual system encodes spatial average properties, such as average orientation, average size, and average numerosity along single dimensions, there has been no investigation of whether the fundamental nature of average representations extends to the temporal domain. Here, we used an adaptation paradigm to demonstrate that the average duration of a set of sequentially presented stimuli negatively biases the perceived duration of subsequently presented information. This negative adaptation aftereffect is indicative of a fundamental visual property, providing the first evidence that average duration is encoded along a single visual dimension. Our results not only have important implications for how the visual system efficiently encodes redundant information to evaluate salient events as they unfold within the dynamic context of the surrounding environment, but also contribute to the long-standing debate regarding the neural underpinnings of temporal encoding.

**Electronic supplementary material:**

The online version of this article (10.3758/s13414-020-02134-8) contains supplementary material, which is available to authorized users.

## Introduction

Our perceptions of the durations of different internal and external events can be influenced by a number of different biological, perceptual, and cognitive biases, raising the question of how we are able to maintain a relatively stable perception of time within our dynamic surroundings. The visual system is not only famously limited in its capacity to process a fraction of information available in a single glance, but also in its ability to process information over time. For example, in addition to being restricted to simultaneously attending approximately four individual objects at any given moment (Pylyshyn & Storm, 1988), observers are also able to process only a fraction of the information in streams of sequentially presented items (e.g., the “attentional blink”; Raymond et al., 1992). In contrast to the large literature concerned with the detailed encoding of individual objects, there are large gaps in our knowledge of what happens to the majority of incoming information that cannot be processed within the scope of the limited-capacity system, and why we nonetheless have the impression of perceiving a detailed, stable, and continuous world.

It has been suggested that the limited-capacity visual system accomplishes our illusion of coherent perception by conceptually integrating occasional detailed samples with an overall interpretation of the “gist” of the world around us and statistical summaries of the remaining areas (Ariely, 2001). By perceptually summarizing or averaging sets of similar objects, the visual system can bypass capacity limitations and provide a compressed representation that is more precise than the individual noisy measurements comprising the set (Alvarez, 2011; Ariely, 2001, 2008), capitalizing on the redundancy inherent in the surrounding environment to most efficiently represent the maximal amount of information (Corbett, 2017; Corbett & Munneke, 2018). Along these lines, our inability to process the details of individual items in a set of objects stands in sharp contrast to our enhanced ability to summarize the overall characteristics of the set. For example, observers are able to accurately determine whether a test circle presented after a set of differently sized circles represents the mean size of the set, but are at chance to determine whether the test circle was a member of the set (Ariely, 2001). This superior ability to represent average versus individual properties has been demonstrated for a broad range of features such as orientation (Parkes et al., 2001), direction of motion (Dakin & Watt, 1997; Watamaniuk et al., 1989), more abstract properties like numeric meaning (Corbett et al., 2006), and even in the auditory domain (McDermott et al., 2013). In fact, no study to date has found evidence to suggest that perceptual averaging can be prevented.

Perceptual averaging has also been heavily implicated in maintaining stable and continuous visual representations over time. For example, even items that are not consciously perceived within a rapidly presented stream of objects are nonetheless included in average representations (Corbett & Oriet, 2011), suggesting summary representations are computed by a qualitatively different, more efficient mechanism than the limited-capacity attentional mechanisms involved in individual object representations. Furthermore, summary statistical representations transfer interocularly, across eye movements, between observer- and world-centered spatial frames of reference (Corbett & Melcher, 2014a), and even guide reaching and grasping movements (Corbett & Song, 2014), allowing statistical context to remain stable as we interact within the surrounding environment. This statistical context builds over time, enabling the visual system to mediate between the needs to maintain stable perception while detecting salient changes (Corbett & Melcher, 2014b).

Evidence for the fundamental nature of summary statistical representations is given by findings that observers experience a negative aftereffect from adapting to the average properties of sets of objects such as their mean size (Corbett et al., 2012), or an overall property that emerges from the set such as numerosity (Burr & Ross, 2008; Durgin, 1995). For example, when observers are adapted to two patches of dots – one with a larger mean size – they perceive a test object presented to the region adapted to the larger mean size patch as smaller than when the same-sized test object is presented to the region adapted to the smaller mean size patch (Corbett et al., 2012). This sort of negative aftereffect of adaptation is a signature of underlying independent mechanisms that selectively encode along a single visual dimension over a limited range (Campbell & Robson, 1968), suggesting that the average properties of sets of objects are encoded as fundamental aspects of visual scenes, like the orientations, sizes, or colors of individual objects.

Although several studies have demonstrated that the visual system encodes the average spatial properties of sets of objects presented over time (Albrecht et al., 2012; Albrecht & Scholl, 2010; Corbett & Oriet, 2011; Dubé & Sekuler, 2015; Hubert-Wallander & Boynton, 2015), and that the most salient frequency dominates estimates of the average frequency of a set of flicking objects (Kanaya, et al., 2018), there has been no systematic investigation of how the visual system may also temporally average duration over sets of spatially similar objects with different durations. It is known that observers adapt to the durations of single items, such that they perceive the duration of a test event as lasting for a shorter amount of time when presented after a relatively longer event versus a shorter event (Heron et al., 2012; Walker et al., 1981; cf. Curran et al., 2016), and that the context of surrounding temporal information can bias the perceived duration of outlier or “oddball” events (Pariyadath & Eagleman, 2007; Tse et al., 2004). Taken together, the fundamental, adaptable natures of average spatial properties and single-event durations suggest that average duration may be similarly encoded along a single visual dimension.

To test this proposal, the present study investigated whether observers can adapt to the average duration of a stream of otherwise identical visual events. We presented participants with two simultaneous streams of items on the left and right of fixation, one stream with a longer average duration than the average duration of the items in the other stream. The adapting streams were followed by two test stimuli, and participants were required to report which test appeared to have the longer duration. Regardless of whether the adapting and test stimuli shared the same spatial properties, observers perceived the test presented in the region adapted to the shorter average duration as lasting longer than the test presented in the region adapted to the longer average duration.

## Experiment 1

In an initial experiment, we tested whether observers experienced a negative adaptation aftereffect of the average durations of two simultaneously presented streams of circles on the perceived durations of two subsequently presented test circles.

### Methods

#### Participants

Twenty-five Bilkent University students were tested in Experiment 1 (11 females, mean age = 20.7 years). All had normal or corrected-to-normal vision and gave informed consent to voluntarily participate in the experiment in exchange for monetary compensation or course credit. All experimental procedures and protocols were approved in accordance with the Declaration of Helsinki by Bilkent University’s Ethics Committee.

#### Task

On each trial, participants adapted to two lateralized streams of sequentially presented circles, and their task was to judge which of the two subsequently presented test circles appeared to remain on the screen longer (had the longer duration). Participants pressed the right arrow on the keyboard if the right test appeared to have the longer duration and the left arrow if the left test appeared to have the longer duration.

#### Apparatus

An HP PC was used to present stimuli on a 21-in. NEC monitor at a resolution of 1,600 × 1,200 pixels and a 60-Hz refresh rate. MATLAB (version 2016b) in conjunction with Psychophysics Toolbox (Brainard, 1997; Pelli, 1997) controlled all the stimulus presentation, response, and data collection functions. Participants were seated approximately 57 cm from the center of the monitor, such that 1° of visual angle corresponded to 37 pixels.

#### Stimuli and procedure

**Fig. 1:**
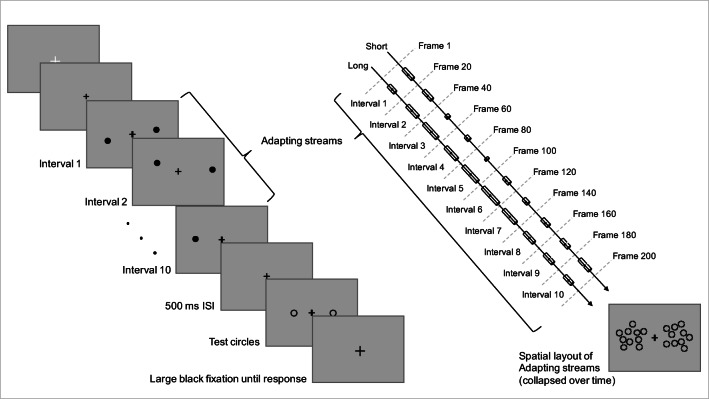
**Left:** Illustration of a trial sequence in Experiment 1 (see Supplementary Video 1 for an additional illustration of a trial sequence). Each trial began with a large white fixation, which turned small and black when participants pressed the space bar to initiate the trial. Next, two adapting streams of ten sequentially-presented, black circles were presented, one on each side of fixation, followed by a 500-ms interstimulus interval (ISI) with only the fixation. Then, two black outline test circles were presented, one in each adapted region. Finally, the fixation enlarged, signaling participants to respond as to whether the left or right test circle appeared to have the longer duration. **Right:** The 200-frame adapting interval was divided into ten equi-temporal intervals and stimuli were temporally presented around midpoints at ten frames to 190 frames, in 20-frame steps, with the durations of the individual circles comprising the adapting streams presented in pseudorandom order on each trial. As illustrated in the spatial layout of Adapting streams (collapsed over time), the spatial location of each individual circle on each trial was pseudorandomly selected without replacement from the ten possible locations

(Figure 1; Supplementary Video 1 (in the Online Supplementary Material) illustrates a sample trial from Experiment 3, with the longer average duration adapting stream on the left): Each trial began with a 1° white fixation cross in the center of the screen until the participant pressed the space-bar to start the trial. Then, the white fixation was replaced by a 0.5° black cross signaling that the trial had started. Participants fixated the central cross and adapted to two streams of ten serially presented 1.5° black, filled circles, one on each side of fixation. Each set of ten circles was composed of two concentric rings: An outer ring of five circles initially positioned around an imaginary circle with a 3° radius at the 0^°^, 72^°^, 144^°^, 216^°^, and 288^o^ positions, and then jittered independently in the x- and y-directions by a random factor between ± 0.135^°^, and a 1.5^°^-radius inner ring of five circles initially positioned and jittered in the same manner. Within each of the two ten-circle patches, we restricted the positions of the circles such that no individual circle was within 0.135^°^ of any other circle in either the x- or y-direction. Each radial array of adapting circles was centered at 8^°^ of eccentricity along the horizontal meridian relative to the center of the screen.

One of the adapting streams had a longer mean duration relative to the other adapting stream. The shorter adapting stream always contained the same ten durations, ranging from two frames to 11 frames in single-frame steps, and the longer adapting stream always contained the same ten durations, ranging from seven frames to 16 frames, also in single steps. Note that half of the individual durations in each adapting stream were unique (the five shorter durations in the shorter adapting stream, and the five longer durations in the longer adapting stream), and half of the durations were identical. In other words, the only differences between the two adapting streams were the five unique individual durations, and therefore the streams’ *overall* shorter or longer *average* durations. Durations were coded as intervals of the monitor’s refresh rate, such that the duration of one frame was approximately 16.67 ms at 60 Hz. Circles with each of the individual durations in each stream were presented sequentially in one of the ten possible locations chosen pseudo-randomly on each trial, such that all of the durations and all of the locations were presented only once during each trial. On each trial, the entire adapting sequence of both streams of circles lasted for 200 frames. The 200-frame interval was divided into ten equi-temporal intervals and stimuli were temporally presented around midpoints at ten frames to 190 frames, in 20-frame steps, such that the stream with the longer average duration did not constantly onset or offset sooner or later than the stream with the shorter average duration and there was no constant rate of flicker or rhythmic-timing in the adapting streams that may otherwise distort their perceived durations (Johnston et al., 2006; Kanai et al., 2006).

Immediately following the two adapting streams, the two test circles were presented; one in each adapted region. The test circles were also 1.5^o^ in diameter. In order to delineate the test circles from the adapting streams, the test circles were presented as unfilled, black outline circles. The exact locations of the test circles on each trial were randomly selected from one of the ten possible adapting circle locations. There were five possible pairs of test durations, two, six, nine, 12, and 16 frames. Unbeknownst to participants, the right test was always presented at a standard duration of nine frames and the duration of the left test was varied pseudo-randomly according to the method of constant stimuli, such that the five test duration pairs were presented an equal number of times on each block. The difference in duration between the left and right tests was -7, -3, 0, 3, or 7 frames, with negative values indicating shorter left test durations. There was an inter-stimulus interval of 500 ms on each trial after the offset of the 200-frame adapting streams. Then, during the following interval of 20 frames, the individual tests were presented at durations pseudo-randomly selected from one frame to ten frames, such that the test with the longer duration did not constantly onset or offset sooner or later than the test with the shorter duration. Immediately after the 20-frame test period, the black fixation was enlarged (1^o^) signaling participants to make their response. The screen remained blank until they responded. Participants were instructed and trained during practice not to respond until the tests had offset and the black fixation cross was enlarged to better ensure their judgments were correctly based on the durations of the tests.

Each participant completed three blocks of 100 trials in each adapting condition (Long adapting stream on Left = LoL; Long adapting stream on Right = LoR) for a total of 600 trials. Within each 100-trial block, the five possible pairs of test durations were each presented 20 times, for a total of 60 trials per test pair in each LoL and LoR adapting condition. The order of the LoL and LoR adapting conditions was counterbalanced over participants. Each experimental session lasted approximately 75 min.

Each participant completed one block of 50 practice trials to familiarize them with the task before beginning the first experimental block. They were presented with written, illustrated instructions at the start of the practice block, and the experimenter ensured they fully understood the task before they were allowed to proceed to the experimental blocks.

While participants did not receive any instructions to pay attention to any aspects of the adapting streams, they were explicitly instructed to remain fixated on the central fixation cross throughout the duration of each trial and to determine which test circle had the longer duration as quickly and accurately as possible on each trial. To help ensure participants remained fixated throughout each trial, we implemented a “red fixation” task. On a random 10% of experimental trials and 50% of practice trials, the fixation cross turned red for 100 ms at a random point during the presentation of the 200-frame adapting streams. In addition to the main task, participants were instructed to press the space bar on the keyboard as soon as they saw the red fixation. Data from these red fixation trials were not included in any analyses. If a participant missed more than five red fixations in a single block, they had to repeat it. In addition, participants had to make their responses within 100–2,500 ms after the offset of the test circles. They were instructed that if they failed to do so more than eight times in a single block, they had to repeat the block. If a participant failed to respond to the red fixation or responded too fast or too slowly on a given trial during the practice block, they were presented with a written warning for 500 ms after the trial. Participants were informed that they could only repeat the practice block one time and one experimental block one time. If they failed the practice block a second time or had to repeat more than one experimental block, they were dismissed from the experiment. No participants in Experiment 1 were dismissed.

### Results and discussion

For each participant, in each adapting condition (LoL and LoR), we calculated the average proportion of “Left test longer” responses for each of the five Left-Right (L-R) test duration differences. We next fit each participant’s averaged data in each condition to two separate logistic functions with lower bounds of 0 and upper bounds of 1 using maximum likelihood estimation. We evaluated the goodness of each fit using deviance scores of the log-likelihood ratio between a fully saturated zero-residual model and the data model, such that a deviance score above the critical chi-square value indicated a significant deviation between the fit and the data (Wichmann & Hill, 2001). The data from two participants in Experiment 1 were excluded from further analyses due to deviance scores above the critical chi-square value (*χ*^2^(4,0.95) = 11.07). The complete statistics for all 25 participants in Experiment 1 are listed in Table 1. The corresponding raw data for all three experiments in the present manuscript are publicly available online via the Open Science Framework (https://osf.io/my4q8/).

**Table 1:** PSE in frames and milliseconds and corresponding deviance score for each participant (ss) in each adapting condition (LoL/LoR) in Experiment 1 (left), Experiment 2 (middle), and Experiment 3 (right)

Experiment 1							Experiment 2							Experiment 3						
LoL				LoR			LoL				LoR			LoL				LoR		
ss	PSE (frames)	PSE (ms)	*x*^2^	PSE (frames(	PSE (ms(	*x*^2^	ss	PSE (frames)	PSE (ms)	*x*^2^	PSE (frames(	PSE (ms(	*x*^2^	ss	PSE (frames)	PSE (ms)	*x*^2^	PSE (frames(	PSE (ms(	*x*^2^
1	0.54	9.00	64.24	-0.12	-2.06	74.03	1	2.02	33.61	39.04	1.14	18.92	50.83	1	1.64	27.39	30.14	1.14	19.07	50.55
2	2.33	38.84	40.14	-1.55	-25.83	62.22	2	-0_08	-14.72	44.99	-0.09	-14.85	40.25	2	1.97	32.76	43.50	3.00	50.02	26.13
3	-3.87	-64.56	28.78	-2.95	-49.14	20.25	3	0.52	5.71	26.00	-0.54	-9.03	14.70	3	0.78	12.95	48.31	-1.51	-25.12	67.02
4	0.55	9.10	18.64	-0.16	-2.73	23.66	4	-0.02	-028	57.65	-1.43	-23.81	67.77	4	-3.33	-55.58	51.77	-3.30	-55.01	45.73
5	-0.45	-7_52	36.36	-0.09	-1.43	45.47	5	-0.58	-9_67	82.77	-1.97	-32.76	65.13	5	0.12	1.94	59.97	0.56	9.27	62.89
6	-0.89	-14.83	42.09	0.25	4.16	54.16	6	0.21	3.43	16.97	-0.49	-5.10	34.75	6	-0.75	-12.45	65.05	0.40	6.67	38.21
7	0.01	0.10	37.07	-0.02	-1137	50.80	7	1_07	17.90	25.14	1.06	17.71	21.44	7	0.87	14.52	53.72	-0.44	-7.32	54.58
8	4.09	68.23	16.48	-0.02	-0.32	19.45	8	2.52	41.96	15.35	2.35	39.25	15.17	8	-0.41	-6.85	43.07	-0.01	-13.54	59.71
9	0.49	8.19	51.53	0.13	2.17	48.54	9	2.77	46.13	42.34	0.17	2.90	62.36	*9	1.27	21.22	49.05	2.15	35.76	36.76
10	2.52	41.95	27.16	1.41	23.46	44.29	10	2.80	46.64	27.47	-0.04	-0.64	33.69	10	-0.48	-5_04	46.11	-2.20	-36.65	54.69
11	3.88	64.64	23.57	0.95	15.79	38.86	11	0.22	3.62	51.16	-1.66	-27.67	59.21	11	0.93	15.51	32.73	0.60	10.06	51.93
12	3.13	52.13	21.46	0.97	16.12	31.04	12	-1.53	-25.54	45.55	-1.35	-22.58	47.42	12	-0.55	-9.20	18.53	-4.77	-79.51	18.44
13	-2.84	-47.37	31.71	-5.15	-85.78	48.08	13	-1.89	-31.53	32.66	-2.90	-48.35	38_15	13	0.87	14.49	14.69	-0.10	-1.59	23.40
14	1.49	24.83	33.33	-0.57	-9_46	65.02	14	2.94	48.99	13.88	1.59	26.54	13.27	14	1.95	32.49	34.63	1.98	32.92	31.18
15	4.24	70.72	12.86	2.03	33.92	19.32	15	1.33	22.24	23.37	1.66	27.69	29.95	15	-1.46	-24.33	73.48	-1.78	-29.60	67.69
16	-1.33	-22.16	46.52	-2.64	-44.07	14.37	16	1.92	31.96	49.43	0.96	15.93	36.47	16	1.32	21.99	27.42	3.12	52.05	24.35
*17	-0.31	-5.23	56.61	-1.80	-30.03	76.94	17	0.54	9.01	45.75	-1.20	-20.01	49.07	17	-0.84	-14.04	37.46	-2.27	-37.92	38.07
18	1.47	24.43	27.41	-1.50	-25.01	50.68	18	1.76	29.41	29.21	2.41	40.21	19.35	18	1.01	16.88	42.93	0.93	15.54	41.32
19	1.47	24.54	16.54	-1.66	-27.75	45.25	19	-0.15	-2.50	42.61	-0.41	-6.91	62.59	19	-0.53	-0.90	72.78	-0.01	-0.22	64.62
20	-0.66	-11.02	32.55	-1.28	-21.35	31.15	*20	-0.69	-11.51	61.41	-1.33	-22.24	69.79	20	0.76	12.61	38.45	-0.51	-8.47	40.25
21	-1.42	-23.74	46.73	-1.97	-32.90	43.61	21	1.25	20.87	21.91	0.64	10.63	31.09	21	0.35	5.83	35.95	0.12	1.92	44.06
*22	2.00	33.29	32.61	2.27	37.87	20.08	22	-1.23	-21.28	22.23	-2.92	-48.76	23.10	*22	-1.40	-23.35	78.02	-1.18	-19.74	66.74
23	1.85	30.89	36.15	0.00	-4.06	42.52								23	2.88	48.01	11.96	0.23	3.82	42.76
24	3.62	60.31	16.23	2.11	35.14	37.13								24	1.14	19.01	29.26	-0.72	-12.05	25.13
25	-2.05	-34.18	29.76	-3.55	-59.13	33.10								25	3.48	57.96	23.58	2.83	47.21	27.29

Asterisks (*) preceding participant numbers signify participants with a logistic fit in one of the adapting conditions having a deviance score above the corresponding critical chi-square value (11.07), and therefore whose data were not included in any further analyses

**Fig. 2:**
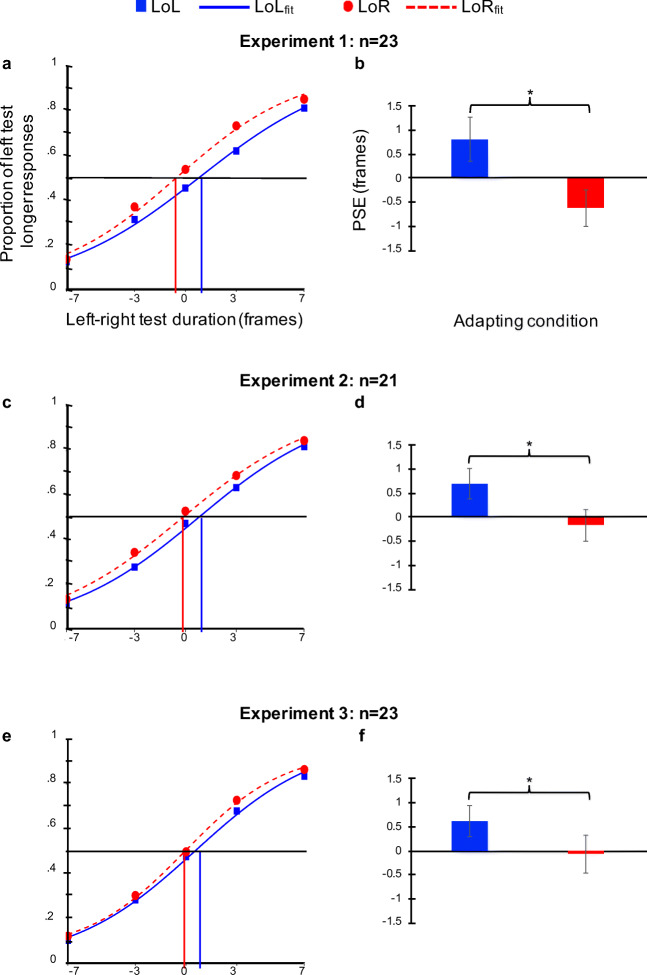
Results (1 frame = 16.67 ms). Left psychometric functions: Based on the logistic fits (lines) for the raw data (points) from each L-R test duration difference, participants perceived the left test circle as lasting longer more often when they were adapted to the stream of circles with the longer average durations on the right (LoR; red dashed lines and circles) than when the adapting stream with the longer average circle duration was presented on the left (LoL; blue solid lines and squares) in (**a**) Experiment 1 (n=25), (**c**) Experiment 2 (n=22), and (**e**) Experiment 3 (n=25). The black horizontal dashed lines represent the 0.5 inflection point on each curve, and vertical dashed lines represent the corresponding PSE values (the difference in duration between the left and right test circles necessary for participants to perceive the left test circle as lasting longer 50% of the time) on the x-axes for each LoL (vertical blue dashed lines) and LoR (vertical red dashed lines) condition. Right column graphs: There was a significant difference between the resultant points of subjective equality (PSEs) for the LoL and LoR adapting conditions in (**b**) Experiment 1, (**d**) Experiment 2, and (**f**) Experiment 3, indicating that participants in all three experiments experienced significant negative aftereffects of adaptation to average duration. Error bars represent the standard error of the mean for each corresponding condition and the asterisks represent significant differences in planned paired t-tests with p < 0.05

Using the data from the remaining 23 participants with significant logistic fits in each of the two adapting conditions, we next calculated the Point of Subjective Equality (PSE); the difference in the duration between the left and right tests needed for each individual to perceive the left test as lasting longer 50% of the time (the 50% inflection point on the corresponding logistic function). As illustrated in the fits for the grand averaged data in each adapting condition, the curve for the LoL adapting condition was shifted rightward relative to the curve for the LoR data (Fig. 2a). A within-subjects paired-samples t-test confirmed significant differences between participants’ PSEs in the LoL and LoR adapting conditions (*t*(22) = 5.24, *p* < 0.001, *d* = 0.71; Fig. 2b).

## Experiment 2

Although the results of Experiment 1 provided strong evidence that participants perceived the duration of a single object as inversely biased by the average duration of the preceding adapting streams of circles, we conducted Experiment 2 to ensure that this negative adaptation aftereffect could not otherwise be explained by lower-level spatial properties of the adapting and test stimuli. Specifically, previous research has shown that stimuli are perceived to last longer when they are more Luminant (Xuan et al., 2007). Therefore, to help ensure that the overall lower luminance on the side of the screen with the longer average duration adapting stream of filled black circles compared to the relatively higher luminance on the side of the screen with the shorter average duration adapting stream of filled black circles in Experiment 1 was not driving the observed difference in the perceived test circle duration, we used unfilled black outline circles in the adapting streams for Experiment 2 (Corbett et al., 2012). In addition, to test whether participants would experience a negative adaptation aftereffect even when the shapes of the elements in the adapting streams were different from the shapes of the test items, we used unfilled black outline squares as the test items in Experiment 2.

### Methods

#### Participants

Twenty-five Bilkent University students were tested in Experiment 2 (11 females, mean age = 20.2 years). All had normal or corrected-to-normal vision and gave informed consent to voluntarily participate in the experiment in exchange for monetary compensation or course credit. One participant was dismissed from the experiment after failing two experimental blocks, and this participant’s data were excluded from any analyses. All experimental procedures and protocols were approved by Bilkent University’s Ethics Committee.

#### Task, apparatus, stimuli, and procedure

All aspects of the experimental design and procedure in Experiment 2 were identical to those used in Experiment 1, with the exceptions that the adapting stimuli were now 1.5^o^ black outline circles and the test stimuli were now 1.5^o^ black outline squares.

### Results and discussion

As in Experiment 1, we fit the raw data for each participant in each adapting condition to two separate logistic functions, and then assessed the goodness of these fits. One participant’s data was excluded from further analysis because the individual pressed the left arrow response key on every trial, and another participant’s data was excluded because the individual alternated left and right button presses over consecutive trials. The complete statistics for the remaining 22 participants in Experiment 2 are listed in Table 1. An additional participant’s data was excluded from further analyses due to deviance scores above the critical chi-square value (*χ*^2^(4,0.95) = 11.07). Using the data from the remaining 21 participants with significant logistic fits in each of the two adapting conditions, we calculated the PSEs as in Experiment 1. Also as in Experiment 1, the curve for the grand-averaged data in the LoL adapting condition was again shifted rightward relative to the curve for the LoR condition (Fig. 2c), and a within-subjects paired-samples t-test revealed significant differences between participants’ PSEs in the LoL and LoR adapting conditions in Experiment 2 (*t*(20) = 4.36, *p* < 0.001, *d* = 0.575; Fig. 2d).

## Experiment 3

The orders of the durations of the stimuli in the adapting streams in Experiments 1 and 2 were determined pseudo-randomly on each trial, such that the longer average duration adapting stream more often began and ended with a longer duration circle than the shorter average duration adapting stream. However, given that previous investigations have demonstrated primacy and recency effects on perceptual averaging such that summary representation of spatial properties like average location, size, facial expression, and motion computed over time do not incorporate all items equally (Hubert-Wallander & Boynton, 2015), we conducted a final experiment to ensure that the effects observed in the first two experiments could not be accounted for solely by the durations of the first or last stimuli in the adapting streams.

### Methods

#### Participants

Twenty-five Bilkent University students were tested in Experiment 3 (16 females, mean age = 20.6 years). All had normal or corrected-to-normal vision and gave informed consent to voluntarily participate in the experiment in exchange for monetary compensation or course credit. All experimental procedures and protocols were approved by Bilkent University’s Ethics Committee.

#### Task, apparatus, stimuli, and procedure

All aspects of the experimental design and procedure in Experiment 3 were identical to those used in Experiment 2, except: (1) the order of the circles in the adapting streams were restricted on each trial such that the durations of the five circles presented in the middle of the streams were pseudo-randomly drawn from the five longest durations that were only used in the long adapting set and the five shortest durations that were only used in the short adapting set, and (2) the durations of the first two or three (determined at random on each trial) and the last three or two circles in each adapting stream were pseudo-randomly drawn from the remaining five overlapping durations that were common to both the long and short adapting sets.

### Results and discussion

We again fit the raw data for each participant in each adapting condition to two separate logistic functions. The complete statistics for the 25 participants in Experiment 3 are listed in Table 1. Two participants’ data were excluded from further analyses due to deviance scores above the critical chi-square value (*χ*^2^(4,0.95) = 11.07). We then calculated the PSEs using the data from the remaining 23 participants with significant logistic fits in each of the two adapting conditions. As in both previous experiments, the curve for the grand-averaged data in the LoL adapting condition was shifted rightward relative to the curve for the LoR condition (Fig. 2e), and a within-subjects paired-samples t-test revealed significant differences between participants’ PSEs in the LoL and LoR adapting conditions in Experiment 3 (*t*(22) = 2.385, *p* = 0.026, *d* = 0.4; Fig. 2f), confirming that participants experienced a negative adaptation aftereffect that could not be accounted for by the first or most recent stimuli in the adapting streams. As a final test for evidence of primacy or recency effects, an independent samples t-test comparing the differences between participants’ PSEs in the LoL and LoR conditions (PSE_LoL_ – PSE_LoR_) in Experiment 2 to the differences in Experiment 3 participants’ LoL and LoR PSEs revealed no significant difference (*t* = 0.5, *p* = 0.627) between the magnitude of the aftereffect in these two experiments with identical methods except for the order of the longest and shortest duration stimuli in the adapting streams.

## General discussion

The present results provide consistent evidence that observers experience a negative adaptation aftereffect to the average duration of a sequentially presented set of visual events that cannot be accounted for by low-level differences in luminance (Experiment 2), shape (Experiment 2), or the duration of any single event (Experiment 3). As adaptation is a signature of independent neural mechanisms that are selectively sensitive over a limited range (Campbell & Robson, 1968), these results significantly advance our understanding of how the visual system internally represents the external environment by explicitly encoding the average duration of events along a single dimension. Importantly, whereas all previous related studies of temporal perception have been concerned with single or homogeneous durations, our results are the first to demonstrate perceptual averaging over sets of spatially similar yet temporally different sets of objects.

Our results support previous proposals that the visual system perceptually averages to allow for an efficient means of encoding the massive amount of information that cannot be explicitly attended and encoded (Alvarez, 2011; Ariely, 2001). Whereas there is a growing literature regarding the fundamental nature of this sort of perceptual averaging of spatial properties such as mean size (Corbett et al., 2012) and numerosity (Burr & Ross, 2008; Durgin, 1995), we demonstrate the first evidence that this sort of statistical compression extends to the purely temporal domain such that observers can represent the average duration of a set of spatially and temporally distributed objects. Building on previous findings that individual duration can bias the perceived duration of subsequently presented items (Heron et al., 2012; Walker et al., 1981), our results further demonstrate that the average duration of a set of objects can also be encoded as a single percept to bias the perceived duration of future events. This sort of efficient representation allows the limited-capacity visual system to evaluate the handful of salient individual events that can be explicitly encoded in each glance within the average temporal context of the massive amount of other visual information in the surrounding environment.

The present findings of duration averaging can be interpreted within the context of outstanding debates in the broader perceptual averaging literature regarding the mechanisms responsible for encoding average representations in any domain. One possibility is that individual elements are automatically processed in parallel, with an average representation formed during early stages of processing such that information about individual items is discounted (Chong & Treisman, 2005). It is also possible that a few individual elements are subsampled, then possibly averaged or otherwise combined or used during later stages of processing (Myczek & Simons, 2008). Both of these accounts could be supported by multiple channels tuned to individual scales. However, the subsampling account is less likely to explain the present results, as subsampling in the present investigation would likely occur in-line with primacy and/or recency effects, and a negative adaptation aftereffect was still observed when the beginning and ends of adapting streams contained the same five durations (Experiment 3). Another possibility is that sets of similar objects are processed holistically without encoding any of the individual items in a manner qualitatively distinct from that in which individual items are represented (Ariely, 2001). Although this type of processing could potentially be accomplished by broadly tuned channels that average out more fine-grained information, it could also be carried out by combining information from multiple individual channels during later stages of processing or by relying on subsamples from individually tuned channels. Despite a lack of consensus about the nature of the mechanisms responsible for perceptual averaging in any domain, there is widespread agreement that such representations are formed at multiple levels of the visual information processing hierarchy (see Alvarez, 2011, and Cohen et al., 2016, for expanded discussions), and need not be consciously accessible to shape and maintain stable perception as we interact within the surrounding environment (Corbett & Melcher, 2014a, 2014b; Corbett & Song, 2014). Along these lines, the temporal aftereffects observed in the present investigation further suggest that perceptual averaging implicitly shapes perception throughout the course of information processing.

In addition to increasing our understanding of how the visual system efficiently encodes the overwhelming amount of incoming information from moment-to-moment, the present results also contribute to the long-standing debate regarding the neural basis of time perception. In line with previous studies demonstrating adaptation aftereffects to single durations (Heron et al., 2012; Walker et al., 1981; c.f. Curran et al., 2016), the present adaptation aftereffects to average durations can be taken as converging evidence for channel-based encoding systems comprised of populations of neural units selectively tuned over ranges of durations. The negative adaptation aftereffect to mean duration reported here also provides a plausible mechanism for models such as Bayesian performance-optimizing model (Jazayeri & Shadlen, 2010), which proposes that the underlying distribution of samples is taken into account such that the system incorporates knowledge about temporal uncertainty to adapt timing mechanisms to the temporal statistics of the environment. Building from recent findings that the visual system accumulates information about the shape of the probability distribution of natural statistics inherent in the surrounding environment (Chetverikov et al., 2016), future studies are necessary to further explore how the visual system similarly relies on the prior probability distribution of temporal information. In addition, investigations of whether summary representations of average duration transfer across different spatial frames of reference in a similar manner to single durations (Li et al., 2015) will further our understanding of when different temporal properties are extracted during the course of sensory information processing. Along these lines, given findings that the spatial spread of single duration adaptation aftereffects is proportional to the size of the adapting stimulus (Fulcher, et al., 2016), it is possible that the aftereffect observed in the present study using sequentially presented and spatially distributed stimuli resulted from an averaging mechanism reading the output of multiple duration channels scattered across the entire adapted region. Given that related studies using adaptation to single durations point to intraparietal sulcus as a potential candidate for duration encoding (Hayashi et al., 2015) possibly during intermediate stages of processing (Li et al., 2017), future work taking advantage of the mean duration adaptation paradigm introduced in the present study not only promise to further uncover the neural underpinnings of temporal perception, but to increase our understanding of the neural substrates involved in encoding average properties of the surrounding environment at multiple levels in the visual hierarchy.

Building on the present findings suggesting that average duration is encoded as a fundamental property of visual information, future investigations are warranted to better understand whether this sort of duration adaptation is localized within retinotopic coordinates, as has been demonstrated for adaptation to oscillating motion or flicker (Johnston et al., 2006; Kanai et al., 2006), or whether adaptation to average duration transfers across eye movements and spatial frames of reference, as has been demonstrated for adaptation to average spatial properties such as mean size (Corbett & Melcher, 2014a). Similarly, an investigation of whether the durations of multiple sets of objects can be simultaneously averaged is necessary to better understand how the visual system may summarize the durations of different sets of objects that are distributed throughout the same location. In the spatial averaging literature, the average properties of two subsets of objects can be represented, but not with the same precision as the corresponding average property of the entire set (Brand et al., 2012). Oriet and Brand (2013) have even demonstrated that it is not possible to prevent the sizes of objects in an irrelevant subset from being included in the average representation the coincident subset. These findings suggest that perceptual averaging in the spatial domain occurs preattentively, before subsets can be explicitly selected. Determining whether the same preattentive patterns hold for averaging duration will greatly contribute to our understanding of how the visual system maintains our perceptions of spatiotemporal stability while mediating the needs to detect salient changes and events within this context.

Overall, the present findings provide the first demonstration that summary statistical representations extend to temporal aspects of sets of objects, such that the visual system extracts average representations of the temporal dynamics of the surrounding environment. In complement to statistical representations of the average spatial properties of sets of objects, such temporal summary statistical representations allow the limited capacity visual system to efficiently evaluate ongoing events as they unfold within the temporal context of the dynamic surrounding environment.

## Electronic Supplementary Material

ESM 1 (MOV 610 kb)

## Data Availability

All data have been made publicly available via the Open Science Framework, and can be accessed at https://osf.io/my4q8/). The experiment and analysis code are also available to individuals upon request (contact jennifer.e.corbett@gmail.com).
